# More Than Money: Experienced Positive Affect Reduces Risk-Taking Behavior on a Real-World Gambling Task

**DOI:** 10.3389/fpsyg.2018.02116

**Published:** 2018-11-05

**Authors:** James Juergensen, Joseph S. Weaver, Christine N. May, Heath A. Demaree

**Affiliations:** ^1^Department of Psychology, Youngstown State University, Youngstown, OH, United States; ^2^Department of Psychology, University Center, Saginaw Valley State University, Saginaw, MI, United States; ^3^Department of Psychology, Springfield College, Springfield, MA, United States; ^4^Department of Psychological Sciences, Case Western Reserve University, Cleveland, OH, United States

**Keywords:** risk-taking, affect, mood-maintenance, house money, break even

## Abstract

Previous research indicates that when people participate in multi-trial games of chance, the results of previous trials impact subsequent wager size. For example, the “house money” and “break even” effects suggest that an individual’s risk-taking propensity increases when financially winning *or* losing during a gambling session. Additionally, the “mood maintenance hypothesis” and affect regulation hypothesis suggest that people in positive and negative affective states are less and more likely to gamble than when in neutral affective states, respectively. In the present study, participants completed a series of trials on three computerized slot machines with varying expected values (EV; −10, 0, +10%) of return on investment, and they were paid a percentage of their final bankrolls in real money. Although results did not support the “house money” or “break even” effects, the “mood maintenance hypothesis” was robustly supported in all EV conditions. This is some of the first evidence supporting this theory using an ecologically valid, real-money gambling task.

## Introduction

Although risk-taking behavior has been investigated for centuries, most early work focused on the logical reasoning underlying an individual’s decision to reject or accept a single proposed gamble (e.g., Bernoulli, 1738). For example, a gamble should be rejected/accepted when the expected utility – the product of the payoff (or “jackpot,” J) and the probability of winning (P) – is lesser/greater than the wager amount (W) (e.g., Bernoulli, 1738; Edwards, 1954). More recent findings have found that people often make decisions counter to expected utility theory, however, and these findings have garnered significant attention. For example, as part of their famous Prospect Theory, Kahneman and Tversky found that people often become risk-seeking on gambles with lower P and higher J and risk-averse on gambles with higher P and lower J (despite W = P^∗^J for all proposed gambles) (Kahneman and Tversky, 1979; Tversky and Kahneman, 1992). Discrepancies between actual decisions involving risk and those predicted by expected utility theory have been increasingly explained by better appreciating the role of emotions on decision-making.

### The Various Influences of Emotion on Risk-Taking Behavior

The first purpose of the present research was to determine how one’s affective state influences risk-taking using an ecologically valid, multi-trial game of chance. Over the past couple of decades, the role of emotion in decision-making processes has been increasingly studied (e.g., Bechara et al., 1994; Schwarz, 2000; Loewenstein et al., 2001; Shiv et al., 2005). One theory especially relevant to risk-taking is the “mood maintenance hypothesis,” which posits that people experiencing positive affect are less likely to take part (or participate to a lesser extent) in risk-taking behaviors compared to people in a neutral affective state (Isen et al., 1988; Nygren et al., 1996; Hermalin and Isen, 2000). Simply stated, this is because people do not want to “risk” their positive affective state in addition to their wager. For example, Nygren et al. (1996) reported that, compared to students who received nothing, undergraduate participants who were greeted warmly and given a small bag of candy were subsequently less likely to wager the course credit that they were about to earn for their experiment participation.

Whereas experienced positive affect has been shown to reduce risky behavior, experienced negative affect has been associated with increased risk-taking behavior (e.g., Raghunathan and Pham, 1999; Lerner and Keltner, 2000). An affect regulation hypothesis suggests that individuals attempt to alleviate the experienced negative affect with the desired (but less likely) outcome of the risky behavior (e.g., Raghunathan and Pham, 1999). That is, individuals in a negative affective state may be more susceptible to riskier behavior because their attention is biased toward the unlikely positive outcome rather than the more likely negative outcome (e.g., Rothermund, 2003; Rothermund et al., 2008).

The second purpose of the present research was to determine how previous outcomes influence future risk-taking behaviors. Two major theories have been developed to help explain this relationship. Dubbed the “house money effect” (Thaler and Johnson, 1990; Post et al., 2006), participants in previous studies became risk-prone after just experiencing a win. When winning during a series of gambling trials, the profit may be considered “house money,” and participants subsequently may feel that they can risk more. Conversely, according to the *Break-Even Effect*, people become more risk-prone when they are losing money, presumably because they have the desire to win back their money. Taken together, prior work suggests that people may show altered risk-taking preferences as a result of previous outcomes and/or emotions, and this is some of the first research to investigate these effects simultaneously.

The vast majority of financial risk-taking research has assessed people’s *imagined* preferences on *single-trial* (i.e., “one-shot”) gambles (e.g., De Martino et al., 2006). This is incongruent with real-world risk-taking experiences, though, especially when it comes to many of the addictive properties associated with risk-taking behaviors. When encountering risk-taking situations in real-life settings (e.g., slot machines), gambles are typically repeated, and choices often lead to financial and emotional outcomes. To improve ecological validity, the present research assesses the role of emotion on risk-taking preferences using a multi-trial task in which wins and losses are actually realized. Thus, using a more generalized task, the present research was designed to improve the understanding of the ways in which both previous outcomes and felt emotion simultaneously impact risk-taking behavior.

### The Value of Cognitive Affective Slots Experiments (CASE)

Besides being able to measure emotional responses to actual wins/losses and risk-taking behaviors, the game tasks used in the present research are unlike the “forced-choice, one-shot” gambles that have often been used in previous research. In others’ tasks, participants typically must choose between a sure-thing like W and a gamble like P^∗^J, where they often receive no outcome. Hence, the choices in those experiments reflect preferences for expected utility. In the present research, we use a computer program designed in-house called the Cognitive Affective Slots Experiments (CASE) (for more information on the CASE, see Demaree et al., 2008). The CASE involves “free-choice, repeated gambles” in which participants select a free parameter (W) in a series of trials, and the outcomes of all trials are received. Thus, the choices made on the CASE reflect preferences for both expected utility as well as experienced utility, and are more similar to many real-world risk opportunities seen in the investment and casino industries. Moreover, participants may be paid in cash at the conclusion of the study based on their performance on the CASE, adding to the ecological validity of this task.

Furthermore, in concordance with the suggestion by Demaree et al. (2012) to broaden the coverage of “risk space,” three *P* = 0.50 (i.e., “coin flip”) versions of the CASE were played with expected values (EV) of −10, 0, and +10%. The impact of altered EV on risk-taking behavior has been rarely investigated, and including these three EV conditions made this possible. Expected utility theory (e.g., Edwards, 1954) and a well-known model of economic rationality in repeated gambling, called proportional betting (also known as the Kelly Formula, Kelly, 1956; Cover and Thomas, 1991; Poundstone, 2005), show that the optimal wager amount in both the EV = −10% and EV = 0% conditions is 0% of one’s bankroll, B (i.e., always $0.00). For repeated positive EV gambling opportunities, the Kelly Formula shows that one should consistently wager a fraction of one’s B equal to (edge/odds), which can be calculated as eP/(e + 1-P). For example, in the EV = + 10% game, the optimal %B wagered would be calculated as 0.1^∗^0.5/(0.1 + 1.0–0.5) = 8.33%. By including a condition in which participants should be wagering something, the present study allows for an examination of whether past outcomes and different emotional states cause people to become sub-optimally risk-averse or risk-prone.

### Current Study

Using the CASE, the present research was designed to determine if: EV impacts risk-taking (Hypothesis 1); positive affect predicts decreased risk-taking behavior (Mood Maintenance Hypothesis, Hypothesis 2); negative affect predicts increased risk-taking behavior (Affect Regulation Hypothesis, Hypothesis 3); W increases as B increases from the initial starting amount (House Money Effect, Hypothesis 4); and W increases as B decreases from the initial starting point (Break-Even Effect, Hypothesis 5). Specifically, the following hypotheses were made:

(1) Participants will, on average, become increasingly risk-seeking and earn more money as the game conditions became more favorable (i.e., from EV = −10% to EV = 0% to EV = + 10%).
(2) The magnitude of one’s positive affective state will predict decreased risk-taking behavior.
(3) The magnitude of one’s negative affective state will predict increased risk-taking behavior.
(4) As the magnitude of one’s B increases above the initial B, increased risk-taking behavior will be observed.
(5) As the magnitude of one’s B decreases below the initial B, increased risk-taking behavior will be observed.

## Materials and Methods

Forty-four undergraduate students were recruited from a Midwestern University. This study was approved by an Institutional Review Board, and participants received course credit and 5% of their final bankroll in cash for their involvement in the experiment.

### Procedure

Upon entering the lab, participants were informed about the procedures of the experiment and provided written consent if they agreed to participate. Participants then completed three different versions of a gambling task created in-house. For each set of gambling trials, participants were given a fifty-dollar bankroll with which to wager and were informed that, at the end of the experiment, they would be paid 5% of their final bankroll in cash. After receiving payment following completion of all three versions of the gambling task, participants were debriefed and thanked for their participation.

### Risk-Taking Measure

#### General Overview

The present study used the Cognitive-Affective Slot Experiments (CASE, Demaree et al., 2008, 2012; Juergensen et al., 2014). The CASE is similar to a slot machine, except that it also assesses participants’ self-reported emotional states after each outcome. Participants were first given instructions about how to play the game and were informed that, for each trial, there was an equal chance of winning or losing. All participants began by completing a version of the CASE game that was “fair” (EV = 0; i.e., the vigorish was set to 0.0%). Subsequently, participants completed, in a counter-balanced order, two additional CASE games, one with a positive EV (+10%) and one with a negative EV (−10%). For example, a $10 wager in the positive EV condition would have an equal chance of resulting in either an $11 win or a $10 loss, compared to a $10 loss or $9 win in the negative EV condition. Before each version of the CASE, participants were informed of the EV of the gambles they were about to experience.

**FIGURE 1:**
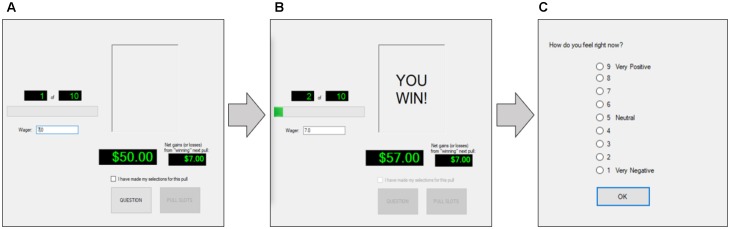
Sequence of events for the CASE. **(A)** Participant enters wager size. **(B)** Participant learns outcome and sees change in bankroll. **(C)** Participant indicates his or her current affect.

For all three versions of the CASE, participants began with a bankroll of $50 with which to wager and were asked to play for 10 trials or as long as they had money in their bankrolls. Participants were informed that, at the conclusion of the experiment, they would be paid 5% of their final bankroll for each CASE game (i.e., if someone didn’t wager at all during the three CASE games, s/he would walk away with [$50 + $50 + $50]^∗^5% = $7.50; if someone had $50, $90, and $145 in their bankrolls at the end of the three CASE games, s/he would walk away with [$50 + $90 + $145]^∗^5% = $14.25). The CASE interface always displayed how much money the player had (B), the current trial (out of 10), and the amount they would win (J) given their current wager amount. The procedure for playing the CASE was as follows: STEP 1, the participant typed in the amount of their wager (from 0 to amount in bankroll) and saw the jackpot amount based on the wager selected; STEP 2, after checking a box indicating that they had made their final wager selection (until they checked this box, they were allowed to alter their wager amount), they then pressed the “Play!” button, learned the outcome (“You lose” or “You win”), and saw their updated bankroll amount; STEP 3, after learning whether they won/lost the gamble, participants answered the question, “How do you feel right now?” using a 9-point Likert scale from 1 (extremely negative) to 9 (extremely positive). The trial number was advanced by 1, and the participant began STEP 1 again. This process continued until Trial 10 was completed or when the participant ran out of money (i.e., B = 0). Figure 1 summarizes these steps.

Please note that, before and after each trial, participants were also asked to predict their emotional response to a possible future win and loss, as well as their actual emotional response to the just-realized outcome. Due to their complexity, these data will be reported elsewhere.

**Specifics; Step 1**: Prior to beginning any version of the CASE, participants were told the probability of winning (i.e., 50% chance of a win/loss) as well as the EV (−10, 0, +10%) of the upcoming trials. These remained constant for all 10 trials of each CASE game. For all versions of the CASE, the jackpot amount for a win was determined by the wager amount, selected by the participant. (Jackpot – Wager) was displayed on the computer monitor during Step 1. In the EV = 0 game, the value of the jackpot equaled (W/P). For the −10, 0, and +10% games, J equaled 0.9W/P, W/P, and 1.1W/P, respectively. Regardless of CASE game played, the net effect of losing a trial was always –W.

**Specifics; Step 2**: After pressing the “Play!” button, the outcome (win or loss) was displayed and the bankroll was altered to display the amount won or lost. (For trials on which the player wagered $0, the screen read “No play” and the trial number advanced by 1).

**Specifics; Step 3**: Following Step 2, the player was asked: “How do you feel right now?” Answers were provided using the Likert scale described previously. (As in Step 2, this question was not asked if the player wagered $0.) The player’s response was taken as a measure of experienced affect, and the trials continued until trial 10 was completed or until cash reserves were depleted (i.e., B = 0). The actual wins and losses experienced during the game were determined randomly by the computer, based on the probability of the game (i.e., participants had a 50% chance of experiencing a win or a loss).

### Data Generated

#### Change in Wager (ΔW)

Consistent with previous research using W as a measure of risk-taking (e.g., Gneezy and Potters, 1997; Demaree et al., 2008), ΔW is the measure of change in risk-taking from one trial to the next. ΔW is defined as W_T_ – W_T−1_, where T = Trial number (1–10). Positive numbers indicate increases in risk-taking (increased wager size), whereas negative numbers indicate decreased risk-taking. ΔW is necessary to determine whether path-dependent risk taking is influenced by outcome and affect experienced on previous trials.

#### Proportion of Bankroll Wagered (PBW)

As wager size will necessarily be capped by the amount of B available on any given trial for each participant, proportion of bankroll wagered (PBW) ranges from 0 to 1 for each participant on each trial. Such a standard measure may allow for a better comparison of risk-taking behavior across participants.

#### Change in Proportion of Bankroll Wagered (ΔPBW)

The change in PBW from one trial to the next is a secondary measure of the change in risk-taking, much like ΔW. However, unlike ΔW, ΔPBW does not depend on the amount of B available from trial to trial and allows for a better comparison of change in risk-taking behavior across participants.

#### Previously Experienced Affect (A_T-1_)

A_T__-1_ represents self-reported affect on the previous trial. Scores greater than 5 (neutral) indicate just-experienced positive affect and scores lesser than 5 indicate just-experienced negative affect.

## Results

In total, 44 participants (25 females, 19 males) with a mean age of 19.36 (*SD* = 1.22) years played all 3 versions of the CASE game. They all began by playing the EV = 0% game (438 total trials) and then, in a counterbalanced order, played the EV = +10% game (440 total trials) and the EV = −10% game (433 total trials). Given the nested nature of the data and the varied number of trials completed by participants, mixed-effects linear modeling was used to test all hypotheses using the lme4 packaged (Bates et al., 2015) for R, a statistical computer program (R Core Team,  2017). The Satterthwaite method was used to estimate the error variance degrees of freedom to determine significance values of models and effects (Kuznetsova et al., 2017).

### Hypothesis Testing

#### Hypothesis 1

**FIGURE 2:**
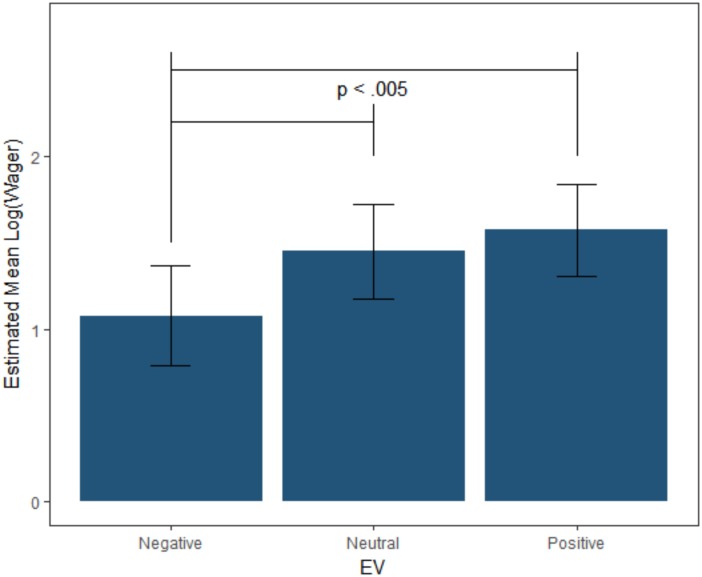
Fixed effect estimates and confidence intervals for the log transformed bankroll wagered in each CASE game. Individuals tended to wager more in the neutral and positive games than the negative game.

To determine if people generally altered risk-taking across the EV_−10%_, EV_0%_ and EV_+10%_ versions of the CASE, a mixed-effect linear model was tested that predicted log-transformed W from EV as a fixed-factor, allowing the slopes to vary across participants as a random effect. Log-transformed values were used to help adjust for the positive skew in wagers. As hypothesized, the expected value of the game significantly predicted wager amount [*F*_(3,41,__984)_ = 49.647, *p* < 0.001]. Planned Tukey contrasts revealed that wagers were significantly higher in the EV_+10%_ (*M* = $8.80, *SD* = $10.94) and the EV_0%_ (*M* = $7.29, *SD* = $8.76) compared to the EV_−10%_ game (*M* = $5.13, *SD* = $6.96; *p*s < 0.001). The EV_+10%_ and EV_0%_ games were not significantly different (*p* = 0.398) (see Figure 2).

A similar model was constructed that predicted log-transformed PBW from EV. As with W, PBW differed by CASE game [*F*_(3,41,__729)_ = 128.92, *p* < 0.001]. Planned Tukey contrasts revealed the same pattern among EV games as was found with W. The EV_+10%_ (*M* = 0.164, *SD* = 0.187) and the EV_0%_ (*M* = 0.173, *SD* = 0.201) games resulted in higher PBW than the EV_−10%_ (*M* = 0.125, *SD* = 0.187) with the EV_+10%_ and the EV_0%_ games not being significantly different (*p* = 0.94).

To assess how participants performed on the 3 versions of the CASE, a repeated measures ANOVA across the 3 EVs was conducted using B after Trial 10 (or whenever the game ended, if the participant ran out of money) as the DV. As anticipated, the expected value of the game significantly predicted ending bankroll [*F*_(2,__86)_ = 12.76, *p* < 0.001]. Planned comparisons using Tukey HSD revealed that final B in the EV _+10%_ game (*M* = $70.86, *SD* = $44.12) was significantly greater than after the EV_0%_ (*M* = $48.41, *SD* = $36.88; *p* < 0.005) and the EV_−10%_ game (*M* = $38.81, *SD* = $20.60, *p* < 0.001). The latter two versions of the CASE were not statistically different (*p* > 0.30).

#### Hypothesis 2 and 3

To determine if individuals changed their risk-taking behavior following the experience of positive or negative affect, a mixed-effect linear regression was created in which wager was regressed onto average wager, the absolute value of experienced affect on the previous trial (|A_T-1_|), change in bankroll on the previous trial, and the interaction of change in bankroll and previous affect. Slopes were allowed to vary across participants. Average wager [β = 1.076, *SE* = 0.004, *t*(1116) = 30.197, *p* < 0.001] and the interaction between the absolute value of previously experienced affect and change in bankroll [*β* = −0.035, *SE* = 0.017, *t*(55.10) = −2.108, *p* = 0.0396] were significant predictors. The significant interaction was explored by fitting mixed-effects linear models for winning and losing trials separately.

**FIGURE 3:**
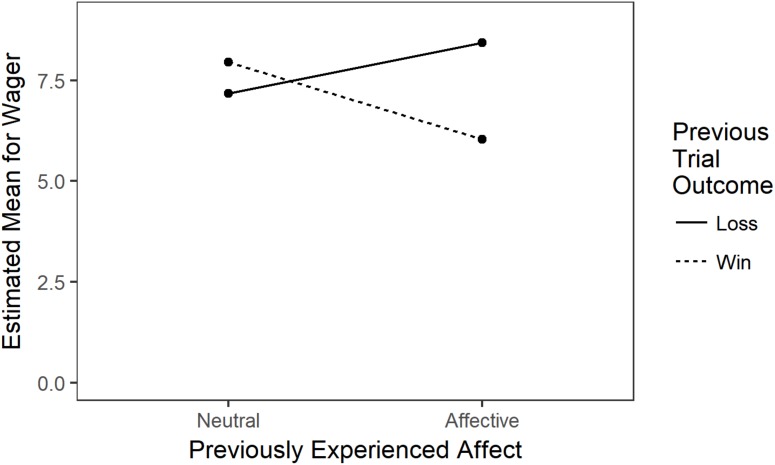
Interaction of previous trial outcome and affect on current trial wager. The effect of affect was statistically significant for winning trials only.

A linear mixed-effects regression for losses regressed wager onto average wager and the absolute value of previously experienced affect, with slopes varying between participants. Average wager remained a significant predictor [β = 1.179, *SE* = 0.062, *t*(41.48) = 19.126, *p* < 0.001] but affect was not a significant predictor [β = 0.315, SE = 0.232, *t*(38.88) = 1.358, *p* = 0.182]. The same model was fit for winning trials. Once again, average wager was a significant predictor [β = 0.918*, SE* = 0.055, *t*(36.47) = 16.65, *p* < 0.001]. Previously experienced affect was also a significant predictor [β = −0.48, *SE* = 0.23, *t*(308.32) = −2.05, *p* = 0.041]. Following wins, individuals tended to wager less as their positive affect increased, but this was not the case following losses. Importantly, a mixed-effects linear regression predicting experienced affect from outcome of trial confirmed that individuals tended to experience positive affect following wins (EM_wins_ = 7.19) and to experience negative affect following losses [EM_losses_ = 3.31; *F*(2,42.99) = 2627.8, *p* < 0.001]. These results support the mood maintenance hypothesis but not the affect regulation hypothesis (see Figure 3).

#### Hypothesis 4

To test the House Money effect, only the data in which B was greater than the initial B amount ($50) at the beginning of the trial were used in all analyses. Due to the differences among scales, the variables were standardized within participant and game for more efficient model fitting and parameter estimation (Bates et al., 2015). The model predicted ΔW from B allowing correlated intercepts and slopes to vary across participants. B trended toward significantly predicting ΔW [β = −0.077, *SE* = 0.043, *t*(37.79) = −1.80, *p* = 0.079].

A parallel mixed-effects model was constructed to predict ΔPBW from percent of initial B (i.e., PB = current B divided by initial B), allowing correlated intercepts and slopes to vary across participants. The variables were standardized within each participant and each game before fitting the model. Percent of initial bankroll was a significant predictor of ΔPBW (β = −0.146, *SE* = 0.043, *t*(38.00) = −3.43, *p* < 0.005]. As individuals’ percentage of initial bankroll increased, they tended to decrease the proportion of bankroll that they wagered. The direction of the relationship between proportion of initial B and ΔPBW was the same as the marginally significant relationship between B and ΔW. These models do not support the House Money effect.

Because bankroll changes as a factor of game (i.e., EV_+10%_ > EV_0%_ > EV_−10%_ ), any effect of bankroll on risk-taking behavior may be accounted for by the type of game being played. To rule out possible confounds, the House Money effect was analyzed within the EV_0%_ game only. Due to the reduction in trials for each participant, a mixed-effect model was unable to be fit and because of the unbalanced data (i.e., differing amounts of trials across participants) a repeated measures approach was also untenable. A linear regression was fit to the standardized average values for each participant. The model predicting ΔW from B was statistically significant [β = 0.466, SE = 0.159, *t*(31) = 2.93 *p* < 0.01] but the model predicting ΔPBW from PB was not significant (*p* > 0.50).

#### Hypothesis 5

The Break-Even Effect was tested in the same manner as the House Money Effect but only the data in which the trial started with B < 50. All variables were standardized within EV and participant before fitting a mixed effects model. The model predicting ΔW from B (allowing slopes to vary across participants) was not statistically significant [*F*(1,48.03) = 1.741, *p* > 0.15]. The model predicting ΔPBW from PB (allowing correlated intercepts and slopes to vary across participants) was significant [β = −0.217, SE = 0.048, *t*(40.69) = −4.485, *p* < 0.001]. This model suggests that, as an individual’s percent of initial bankroll decreased, the proportion of his or her bankroll wagered increased. However, similar to hypothesis 3, the increasing PBW is likely due to the decreasing B as the individuals lose throughout the game.

As with the House Money Effect, we analyzed the data relating to the Break-Even Effect just within the EV_0%_ game. The average values for each participant were calculated and the variables standardized. The linear regression predicting ΔW from B trended toward significance [β = 0.288, *t*(36) = 1.80, *p* = 0.0799] but the regression predicting ΔPBW from PB was not statistically significant (*p* > 0.20).

## Discussion

The present research was designed to assess the role of previous outcomes and experienced affect on risk-taking preferences. Importantly, rather than relying on single and/or hypothetical questions, the present research benefited by collecting self-report data using a relatively real-life, multi-trial assessment tool. Furthermore, participants were informed prior to participation that they would receive financial compensation based on their performance on the risk-taking tasks, thus providing a higher level of ecological validity compared to most experimental paradigms. This is also some of the first research to investigate the impact of altered EV on risk-taking tendencies.

There was a significant difference in risk-taking across EV conditions, such that as EV became more favorable, risk-taking increased. Participants wagered significantly less in the EV-_10%_ game compared to the EV_0%_ game, which was significantly less than the average amount wagered in the EV_+10%_ game. Participants maintained a relatively good strategy in that they wagered the most in the EV _+__10%_. These results provide evidence that participants understood the rules of the game, betting the most money when the odds were in their favor, and the least when the odds were most against them. According to both expected utility theory (Edwards, 1954) and the proportional betting model (also known as the Kelly Formula, Kelly, 1956; Cover and Thomas, 1991; Poundstone, 2005), it would be advisable to not place any wagers in the EV_−10%_ and EV_0%_ games. However, much like the behavior frequently observed in real-world casinos every day, participants continued to place wagers despite being under very obvious negative expected value conditions. This could be a result of how people frame their prospective gambles, as previous research has indicated that people often perceive objectively negative value games as subjectively positive (Rachlin, 1990). Specific to electronic gaming machines, such as the paradigm used in this experiment, individuals often explain their behavior by stating the games are fun and a form of entertainment (Downes et al., 1976; Schellinck and Schrans, 1998). Much like in real-life casinos, it seems evident that participants’ wagers are at least partially dictated by subjective evaluations rather than just focusing on the objective odds. This is some of the first research using multiple EV conditions to measure this type of behavior.

Following winning trials, participants tended to wager less money as their affect increased. This relationship held even when controlling for an individual’s general wager tendency (i.e., average wager). These results strongly support the “mood maintenance hypothesis,” which suggests that people experiencing positive affect are less likely to participate (or at least participate to a lesser extent) in risk-taking behaviors compared to people in neutral affective states (Isen et al., 1988; Nygren et al., 1996; Hermalin and Isen, 2000). People presumably do not want to take unnecessary gambles, simultaneously risking both their positive affect and money. In the present research, because participants generally risked at higher-than-optimal levels, positive affect caused people to have more optimal risk-taking propensities across negative, neutral, and positive EV situations. Although it is possible that positive affect causes people to risk at more optimal levels, we presume that, consistent with the Mood Maintenance Hypothesis, positive affect decreases risk-taking in continuous gambling.

Contrary to our expectations and the affect regulation hypothesis (e.g., Raghunathan and Pham, 1999; Lerner and Keltner, 2000), negative affect on the previous trial did not lead to a reliable increase in risk-taking on subsequent trials on the CASE. We believe that our data are more reliable than those collected in previous work, given that our data are repeated in nature and have direct measures of both affect and risk-taking. These are significant improvements compared to previous related research, which often assessed imagined preferences on single-trial gambles (e.g., De Martino et al., 2006).

Dubbed the “house money effect” (Thaler and Johnson, 1990; Post et al., 2006), participants in previous studies have shown a greater likelihood to participate in risky behavior when winning during a series of gambling trials. When gambling with profit, because the extra capital may be considered “house money,” participants may feel that they can afford to lose more. The results of the current study do not support this effect. Across all EV conditions, the more money participants had relative to their reference point (here, assumed to be the starting capital – $50), the less risk-seeking they became. Stated differently, as B increased (above the starting amount), W and PBW decreased. This is counter to the house money effect. When considering just the neutral EV game, participants did evidence an increase in W as a result of B, but this was not the case for PBW.

The *Break-Even Effect* suggests that people become more risk-seeking when they are losing money (McGlothlin, 1956; Thaler and Ziemba, 1988; Thaler and Johnson, 1990; Coval and Shumway, 2005; Post et al., 2006). Previous research has evidenced that, when experiencing negative emotions, people become more risk-prone (e.g., Raghunathan and Pham, 1999; Lerner and Keltner, 2000). In this study, it was expected that the less capital participants had relative to their reference point, the more risk-seeking they would become. That is, as B decreased (below the starting capital), it was expected that W and PBW would increase. This, however, was only true for PBW. As individuals lost more money, they risked a higher proportion of the bankroll they had remaining. When including only the data from the neutral EV game, results approached but did not meet significance for ΔW and ΔPBW based on B. Taken together, these results do not support the break-even effect.

### Limitations and Future Directions

There were some limitations to the current research paradigm. Because participants were paid only a proportion (5%) of their final bankroll, wager size may have been inflated, as wins/losses were not as meaningful as the actual dollar amount wagered. That said, paying participants even a modest proportion of their bankroll increases the ecological validity compared to paradigms involving no tangible result from wagers (the vast majority of previous research). Moreover, the 3 versions of the CASE in the present study limited participants to only 10 trials per condition, with a maximum of 30 trials total. In real-world gambling settings, there is frequently no finite cap for number of wagers, and gambling is not necessarily limited by one’s bankroll. Future research could involve paying participants a larger proportion of their total bankrolls, including a larger number of wagers, and allowing participants to take out “loans” for further play in order to increase the ecological validity of the paradigm to better approximate the real-world. Regarding the impact of positive affect on risk-taking, future studies could incorporate an affect manipulation procedure in order to strengthen the experiment and also reduce potential confounds.

## Conclusion

The current study utilized the Cognitive-Affective Slot Experiments (CASE, Demaree et al., 2008, 2012; Juergensen et al., 2014) to introduce more ecological validity to the investigation of risk-taking as well as to determine the role of experienced affect in risky decision making. Across three different EV conditions, individuals did behave somewhat rationally, in that they wagered the most in the positive EV condition. However, they repeatedly placed wagers in the negative EV condition, mimicking real-world observations in negative EV casino gambling (e.g., slot machines). The primary finding in this experiment is that when participants reported experiencing greater affect on winning trials, their subsequent risk-taking was reduced. This is some of the first evidence supporting the mood maintenance hypothesis using an ecologically valid, real-money gambling task.

## Ethics Statement

This study was carried out in accordance with the recommendations of the Case Western Reserve University Ethical Guidelines. The protocol was approved by the Case Western Reserve University Institutional Review Board. All subjects gave written informed consent in accordance with the Declaration of Helsinki.

## Author Contributions

JJ was involved in data analyses, writing, and editing the manuscript. CM was involved in writing and editing the manuscript. JW was involved with data analyses, writing, and editing the manuscript. HD was involved with data collection and editing of the manuscript.

## Conflict of Interest Statement

The authors declare that the research was conducted in the absence of any commercial or financial relationships that could be construed as a potential conflict of interest.
